# TLR4 inhibitor TAK-242 attenuates the adverse neural effects of diet-induced obesity

**DOI:** 10.1186/s12974-018-1340-0

**Published:** 2018-11-05

**Authors:** V. Alexandra Moser, Mariana F. Uchoa, Christian J. Pike

**Affiliations:** 1 Neuroscience Graduate Program, University of Southern California, 3641 Watt Way, HNB 120, Los Angeles, CA 90089 USA; 2 Leonard Davis School of Gerontology, University of Southern California, 3715 McClintock Avenue, Los Angeles, CA 90089-0191 USA

**Keywords:** Adiposity, Alzheimer’s disease, Inflammation, Obesity, Toll-like receptor 4, Microglia

## Abstract

**Background:**

Obesity exerts negative effects on brain health, including decreased neurogenesis, impaired learning and memory, and increased risk for Alzheimer’s disease and related dementias. Because obesity promotes glial activation, chronic neuroinflammation, and neural injury, microglia are implicated in the deleterious effects of obesity. One pathway that is particularly important in mediating the effects of obesity in peripheral tissues is toll-like receptor 4 (TLR4) signaling. The potential contribution of TLR4 pathways in mediating adverse neural outcomes of obesity has not been well addressed. To investigate this possibility, we examined how pharmacological inhibition of TLR4 affects the peripheral and neural outcomes of diet-induced obesity.

**Methods:**

Male C57BL6/J mice were maintained on either a control or high-fat diet for 12 weeks in the presence or absence of the specific TLR4 signaling inhibitor TAK-242. Outcomes examined included metabolic indices, a range of behavioral assessments, microglial activation, systemic and neuroinflammation, and neural health endpoints.

**Results:**

Peripherally, TAK-242 treatment was associated with partial inhibition of inflammation in the adipose tissue but exerted no significant effects on body weight, adiposity, and a range of metabolic measures. In the brain, obese mice treated with TAK-242 exhibited a significant reduction in microglial activation, improved levels of neurogenesis, and inhibition of Alzheimer-related amyloidogenic pathways. High-fat diet and TAK-242 were associated with only very modest effects on a range of behavioral measures.

**Conclusions:**

These results demonstrate a significant protective effect of TLR4 inhibition on neural consequences of obesity, findings that further define the role of microglia in obesity-mediated outcomes and identify a strategy for improving brain health in obese individuals.

**Electronic supplementary material:**

The online version of this article (10.1186/s12974-018-1340-0) contains supplementary material, which is available to authorized users.

## Background

The high prevalence of obesity presents a major public health concern since obesity is strongly linked with increased risk for several diseases including type 2 diabetes, cardiovascular disease, and cancer [1]. Importantly, obesity is also associated with adverse effects on the brain and neural function. In humans, obesity is linked with decreases in hippocampal volume and white matter integrity [2–4] as well as with functional consequences that lead to accelerated cognitive decline [5, 6] and increased risk of dementia [7]. In rodent models, diet-induced obesity (DIO) has been demonstrated to impair neurogenesis [8, 9], synaptic plasticity [10, 11], and neural function [12], as well as promote Alzheimer’s disease (AD)-related pathology [13, 14].

Although the mechanisms by which obesity impairs neural health have yet to be fully elucidated, pathways associated with microglial activation are compelling candidates. Obesity is characterized by chronic activation of macrophages in peripheral tissues [15–17] and both microglia and astrocytes in the brain [18–21]. Activated macrophages yield unresolved inflammation in peripheral organs including the adipose tissue [15, 22] and liver [23], whereas activated microglia can drive neuroinflammation in the brain [24, 25]. Neuroinflammation is associated with numerous deleterious effects including reductions in neurogenesis [26] and synaptic plasticity [27] and acceleration of AD [28]. In addition to promoting pro-inflammatory pathways, activated microglia exhibit diverse phenotypes that are characterized by a range of morphological and gene expression signatures [29, 30] and presumed to underlie both beneficial and adverse effects [31, 32]. The pathways that may contribute to the neural effects of obesity remain to be fully defined.

The pattern recognition receptor Toll-like receptor 4 (TLR4) activates signaling pathways that may be particularly important in mediating obesity-associated microglial activation and its consequences. TLR4 stimulation results in downstream activation of at least two key transcription factors: NFκB, which increases expression of pro-inflammatory cytokines [33], and interferon regulatory factor 3, which promotes activated microglial phenotypes that are relatively anti-inflammatory [34, 35]. Thus, TLR4 activation may be expected to yield a range of activated microglial phenotypes. Interestingly, TLR4 binds to and is activated by saturated fatty acids, which are abundant in obesogenic diets and may contribute to obesity-induced increases in inflammation [36–40] and impaired insulin signaling [37, 41]. Prior work has implicated TLR4 signaling as an important regulator of DIO effects on peripheral tissues. For example, mice with either nonfunctional or deleted TLR4 exhibit significant protection against high-fat diet (HFD)-induced glucose dysregulation [42, 43], insulin resistance [44, 45], and peripheral inflammation [46–48], though other studies indicate these mice are not protected against the entire range of metabolic and inflammatory effects of HFD [48, 49]. Pharmacological inhibition of TLR4 also protects mice against HFD-associated adipose inflammation and fibrosis [50] and insulin resistance [51]. Disruption of TLR4 signaling appears to have only modest effects on increases in body weight and adiposity that result from HFD [45, 46, 52, 53].

The potential role of TLR4 signaling in mediating obesity-induced microglial activation and associated neural impairment is unclear. Prior work has implicated TLR4 in pro-inflammatory effects of saturated fatty acids and HFD in hypothalamus, which in turn may regulate diet-induced changes in metabolic function [54–56]. Given that TLR4 is highly expressed in microglia [57, 58], TLR4 signaling pathways are implicated in activated microglial phenotypes, and activated microglia are thought to drive many of the adverse effects of obesity and HFD in hippocampus and other brain regions [10, 59], TLR4 may mediate HFD-induced microglial activation and dysfunction in hippocampus. To address this possibility, we evaluated HFD-induced effects on metabolic, inflammatory, microglial, and neural outcomes in the presence and absence of a pharmacological inhibitor of TLR4 signaling. We report that treatment with a specific TLR4 inhibitor reduced peripheral inflammation and largely prevented both microglia activation and impaired neurogenesis in hippocampus independently of the effects on weight gain and metabolic dysregulation associated with HFD.

## Methods

### Animal procedures

Ten-week-old male C57BL6/J mice were purchased from Jackson Labs (Bar Harbor, ME, USA) and allowed to acclimate to our vivarium facility at the University of Southern California for 2 weeks. Animals were housed under a 12-h light/dark cycle with lights on at 6 AM and ad libitum access to food and water. At 12 weeks of age, mice were randomized to a total of four dietary and drug treatments groups (*N* = 10–14/group). Dietary treatments were either control (CTL; 10% fat; #D12450J, Research Diets, New Brunswick, NJ, USA) or high-fat diet (HFD; 60% fat; #D12492, Research Diets). Drug treatments were either vehicle (0.09% sterile saline) or the TLR4 inhibitor TAK-242 (3 mg/kg in saline; #614316, EMD Millipore, Billerica, MA, USA). Drugs were administered via intraperitoneal (IP) injection 6 days/week. Dosage was based upon a previous study in which TAK-242 delivered at 3 mg/kg via IP injection yielded significant brain levels of the drug that were sufficiently maintained for at least 24 h after administration [60]. Treatments were maintained over a 12-week experimental period, during which body weights were recorded daily and food consumption was measured weekly.

At the conclusion of the experimental period, mice were euthanized with inhalant carbon dioxide and the brains were rapidly removed. One hemi-brain was immersion fixed for 48 h in 4% paraformaldehyde/0.1 M PBS, then stored at 4 °C in 0.1 M PBS/0.03% NaN_3_ until processed for immunohistochemistry. Hippocampus was dissected and snap frozen for subsequent use in RNA extraction, while the remainder of the hemi-brain was snap frozen for subsequent use in protein extraction to examine soluble β-amyloid (Aβ) levels. Blood was collected via cardiac puncture into EDTA-coated tubes and centrifuged to separate plasma, which was stored in aliquots at − 80 °C. Gonadal and retroperitoneal (RP) fat pads were dissected and weighed as measures of adiposity. Both fat pads were snap frozen for subsequent RNA extraction. All animal procedures were conducted under protocols approved by the University of Southern California Institutional Animal Care and Use Committee and in accordance with National Institute of Health standards.

### Body composition

Body composition was determined 1 day prior to euthanization using the Bruker LF90 Minispec (Bruker Optics, Billerica, MA, USA). Mice were placed and loosely restrained inside an acrylic cylinder. The cylinder was placed inside the bore of the magnet, and measurements of fat, lean, and fluid mass percentages were recorded. Animals were returned to their home cages in less than 2 min.

### Glucose, cholesterol, and triglyceride measurements

At weeks 0, 4, 8, and 11, blood glucose readings were measured after overnight fasting (16 h). Blood was collected from the lateral tail vein and immediately assessed for glucose levels using the Precision Xtra Blood Glucose and Ketone Monitoring System (Abbott Diabetes Care, Alameda, CA, USA).

At week 11, glucose tolerance testing (GTT) was performed. First, baseline fasting glucose levels were taken. Mice were then administered a glucose bolus (2 g/kg body weight) via IP injection. Blood glucose levels were recorded from lateral tail vein 15, 30, 60, and 120 min after the glucose bolus. Area under the curve (AUC) was calculated.

Plasma cholesterol and triglyceride levels were measured enzymatically at the conclusion of the experimental period. Commercially available kits for both cholesterol (Total Cholesterol Colorimetric Assay kit, #K603, BioVision, Milpitas, CA, USA) and triglycerides (LabAssay Triglycerides, #290-63701, Wako Chemicals, Richmond, VA, USA) were used following the manufacturers’ protocols.

### Behavioral analyses

All behavioral testing was conducted between the hours of 6 AM and 1 PM. For all behavioral assays, mice were brought into the behavior room and allowed to acclimate for 30 min prior to testing. After each trial, animals were returned to their home cages and the testing arenas were disinfected with 70% ethanol.

Open field and forced swim testing were video recorded and analyzed by a rater blind to experimental treatment groups. Elevated plus maze and spontaneous alternation behavior were scored live. Fear conditioning was recorded using Noldus Ethovision XT software (Leesburg, VA, USA) and the Ugo Basille Fear Conditioning System NG (Gemonia, Varese, Italy).

#### Anxiety and exploratory activity: open field and elevated plus maze (EPM)

Open field test was performed during week 8 of treatments. Briefly, animals were placed into a 40-cm^2^ plexiglass arena and allowed to move freely for 5 min. The arena floor was lightly marked off into 9 squares, with 3 squares along each wall and 1 center square. The following behaviors were recorded: (1) center crossings: the number of times the animal crossed into the center square with both front paws; (2) center time: the amount of time the animal spent with both front paws in the center square; and (3) crossings: the total number of times the animal crossed a line entering a different square.

EPM testing was performed on the day immediately following the open field assay. After being habituated to the room, mice were placed in the center of the EPM, facing a closed arm, and allowed to move freely on the maze for 5 min. The following behaviors were recorded: (1) open arm entries: the number of times the mouse placed both front paws into the open arm; (2) open arm time: the amount of time the animal spent with both front paws in the open arm; and (3) latency to enter the open arm for the first time.

#### Learning and memory: spontaneous alternation behavior (SAB) and fear conditioning

At week 10, SAB was tested in the Y-maze as previously described [61, 62]. Briefly, animals were placed into the long arm and allowed to explore the maze for 5 min. Arm choices were recorded, and behavior was scored as the number of alternations divided by the total number of arm entries.

Fear conditioning was performed over 3 consecutive days beginning 48 h after SAB. On day 1, animals were placed in the conditioning chamber, a box (17 cm × 17 cm × 25 cm) with an electrified grid floor, placed inside a sound attenuated chest (Ugo Basile). White noise was used to block out external sounds. After a 3 min habituation, mice were exposed to 5 tone-and-foot shock pairings that were each placed 3 min apart (20 s tone at 85 dB and 2 kHz, followed by a 20 s trace period, and a 1 sec 1 mA foot shock). Animals were returned to their home cages 1 min after the final tone-shock pairing. Twenty-four hours after training, cued fear conditioning was tested by placing animals back into the chamber but changing the context by altering the pattern of the walls, placing a floor board over the grid floor, and adding a cotton ball with vanilla extract to change the scent of the chamber. After a 3-min baseline period, the tone was played 3 times, but was not followed by the foot shock. Freezing behavior (defined as the absence of all movement except breathing) to the tone and during the 20 s after the tone was recorded. On day 3, 24 h after cued testing, contextual fear conditioning was assessed by placing animals back into the chamber that had the same appearance and odor as it did during training on day 1. Freezing behavior was measured over 8 min. Behavior in the fear conditioning chamber was recorded using Noldus Ethovision XT software.

#### Depression-like behavior: forced swim test (FST)

FST was conducted 1 week after fear conditioning, during week 11, and was the last behavioral assessment. As previously described [61], the animals were placed into a 2-L cylindrical tank (20 cm height × 13 cm diameter) filled with 15 cm of water heated to 23–25 °C. At this depth, neither the feet nor tails of animals reached the floor of the cylinder. Mice remained in the cylinder for 5 min, during which behavior was videotaped from the side of the cylinder. Animals were scored as being immobile if they were making only the movements necessary to keep their head above water. The number of immobile bouts, the total time spent immobile, and the duration of the longest bout of immobility were recorded.

### Immunohistochemistry and quantification

Fixed hemi-brains were completely sectioned at 40 μm in the horizontal plane, using a vibratome (Leica Biosystems, Buffalo Grove, IL, USA). A standard avidin/biotin peroxidase approach using ABC Vector Elite kits (Vector Laboratories, Burlingame, CA, USA) was used to perform immunohistochemistry, as previously described [63]. Every eighth section was processed for ionized calcium binding adaptor molecule 1 (IBA-1), doublecortin (DCX), and bromodeoxyuridine (BrdU). A different initial antigen retrieval step was performed for each antibody, after which the same protocol was followed. For IBA-1 staining, sections were boiled in 10 mM EDTA, pH 6.0 for 10 min, then rinsed in water three times for 5 min each. For DCX staining, tissue was pretreated with 95% formic acid for 5 min, followed by rinsing in TBS. Finally, for BrdU staining, sections were placed in 1% NP40 detergent for 20 min, rinsed in TBS, then incubated in 2 N HCl at 37 °C for 30 min, followed by 10 min in 0.1 M boric acid and rinsing in TBS. Following the various antigen retrieval steps, sections were treated with an endogenous blocking solution for 10 min, then rinsed with 0.2% Triton-X in TBS, 3 times for 10 min each. Tissue was then incubated for 1 h in a blocking solution consisting of 2% bovine serum albumin and 0.2% Triton-X in TBS for IBA-1, plus 2% normal goat serum for BrdU. For DCX, the blocking solution was made up of 3% normal horse serum and 0.2% Triton-X in TBS. Blocked sections were incubated overnight at 4 °C in primary antibody directed against IBA-1 (#019-19741, 1:500 dilution, Wako Chemicals); DCX (#sc-271390, 1:1000 dilution, Santa Cruz Biotechnology, Dallas, TX, USA); or BrdU (#MCA2483, 1:200 dilution, Bio-Rad, Hercules, CA, USA). All primary antibodies were diluted in the respective blocking solution used. On the following day, sections were rinsed and incubated in biotinylated secondary antibody diluted in blocking solution. Finally, immunoreactivity was visualized using 3,3′-diaminobenzidine (Vector Laboratories).

Density and activation states of microglia were determined using live imaging under bright-field microscopy with a × 40 objective (Olympus, BX50, CASTGrid software, Olympus, Tokyo, Japan). As previously described [63, 64], each cell was scored as having either a resting or reactive phenotype. Specifically, resting or type 1 microglia were defined as having spherical cell bodies with numerous thin, branched processes. Both type 2 and 3 microglia were considered reactive: type 2 cells had enlarged, rod-shaped cell bodies with fewer and thicker processes, while type 3 cells were enlarged and had either very few or no processes, or several filopodia. Microglia were quantified in the entorhinal cortex (4 fields/section), subiculum (4 fields/section), CA1 (5 fields/section), and CA2/3 (3 fields/section) across 4 tissue sections for a total of 64 fields and an average of ~ 450 cells per brain. Because increased soma size is a robust indicator of microglial activation [65], we also examined microglial soma size. Images of IBA-1 immunostaining in the CA1 subregion of the hippocampus were digitally captured using an Olympus BX50 microscope and DP74 camera paired with a computer running CellSens software (Olympus). Microglial cell bodies were outlined, and their area was determined using NIH ImageJ 1.50i (US National Institutes of Health, Bethesda, MD, USA).

DCX- and BrdU-immunoreactive cells were also quantified using live imaging under bright-field microscopy with a × 100 oil immersion lens (Olympus). Cells were counted in non-overlapping fields of the subgranular zone and granule cell layer of the dentate gyrus, across 8 sections per animal. Additionally, to examine the relative maturity of DCX-expressing cells, the morphology of their dendritic processes was assessed as previously described [66–68]. Briefly, immature or type 1 cells were defined as having very short or no processes, intermediate or type 2 cells as having processes that extended only within the granule cell layer and do not extend into the molecular layer, and post-mitotic or type 3 cells as having dendrites that extend and branch into the molecular layer or having multiple branches within the granule cell layer. Morphology of DCX-positive cells was assayed across 4 sections per animal, and the relative proportions of type 1, 2, and 3 cells were calculated.

### RNA isolation and quantitative PCR

RNA was extracted from the gonadal fat pads and the hippocampus using TRIzol reagent (Invitrogen Corporation, Carlsbad, CA, USA), following the manufacturer’s protocol. To remove any remaining DNA contamination, the RNA pellet was treated with RNase-free DNase I (Epicentre, Madison, WI, USA) for 30 min at 37 °C after which a phenol-chloroform extraction was performed to isolate RNA. cDNA was reverse transcribed from 1 μg of purified RNA using the iScript cDNA synthesis system (Bio-Rad). The resulting cDNA was used to run real-time quantitative PCR using SsoAdvanced Universal SYBR Green Supermix (Bio-Rad) and a Bio-Rad CFX Connect Thermocycler, as previously described [63]. Both hippocampus and adipose tissue were analyzed for expression levels of cluster of differentiation 68 (CD68), EGF-like module-containing mucin-like hormone receptor-like 1 (F4/80), major histocompatibility complex class II (MHC II), cluster of differentiation 74 (CD74) transcript variant 1, interleukin-6 (IL-6), and interleukin-1β (IL-1β). Additionally, hippocampal tissue was assessed for lipoprotein lipase (LPL) and CD36, as well as for the Aβ clearance and production factors neprilysin, insulin-degrading enzyme (IDE), and β-site APP cleaving enzyme (BACE1). Finally, levels of the cytokine tumor necrosis factor α (TNFα) transcript variant 1 were examined in adipose tissue. Primer pair sequences for target genes are shown in Table 1. All samples were run in duplicate, and PCR products were normalized with corresponding expression levels of β-actin and/or phosphoglycerate kinase 1 (Pgk1) in the brain and succinate dehydrogenase complex, subunit A, flavoprotein (SDHA) in the adipose tissue. The ΔΔ-CT method was used to determine relative mRNA levels. For hippocampal samples, the Ct value of each reference gene (β-actin and/or Pgk1) was subtracted separately from the target genes and the resulting values were averaged and used to calculate fold changes relative to the control-diet, vehicle-treated group. CD68, F4/80, MHCII, CD74, IL6, IL1β, LPL, and CD36 were run with both reference genes and neprilysin, IDE, and BACE1 only with β-actin.

**Table 1:** Target genes for the PCR analyses are listed with their corresponding GeneID number and oligonucleotide sequences for the forward and reverse primers

Target gene	Primer sequence
β-actin *Gene ID: 11461*	Forward: 5′-AGCCATGTACGTAGCCATCC-3′ Reverse: 5′-CTCTCAGCTGTGGTGGTGAA-3′
β-site APP cleaving enzyme (BACE1) *GeneID: 23821*	Forward: 5′-TCGCTGTCTCACAGTCATCC-3′ Reverse: 5′-AACAAACGGACCTTCCACTG-3′
Cluster of differentiation factor 36 (CD36) *GeneID: 12491*	Forward: 5′-TATTGGTGCAGTCCTGGCTG-3′ Reverse: 5′-CTGCTGTTCTTTGCCACGTC-3′
Cluster of differentiation factor 68 (CD68) *GeneID: 12514*	Forward: 5′-TTCTGCTGTGGAAATGCAAG-3′ Reverse: 5′-AGAGGGGCTGGTAGGTTGAT-3′
Cluster of differentiation factor 74 (CD74), transcript variant 1 *GeneID: 16149*	Forward: 5′-CAAGTACGGCAACATGACCC-3′ Reverse: 5′-GCACTTGGTCAGTACTTTAGGTG-3′
EGF-like module-containing mucin-like hormone receptor-like 1 (F4/80) *GeneID: 13733*	Forward: 5′-TGCATCTAGCAATGGACAGC-3′ Reverse: 5′-GCCTTCTGGATCCATTTGAA-3′
Insulin-degrading enzyme (IDE) *GeneID: 15925*	Forward: 5′-TGTTTCCACACACAGGCAAT-3′ Reverse: 5′-ACCTGTGAAAAGCCGAGAGA-3′
Interleukin-1β (IL1β) *GeneID: 16176*	Forward: 5′-GCAACTGTTCCTGAACTCAACT-3′ Reverse: 5′-ATCTTTTGGGGTCCGTCAACT-3′
Interleukin-6 (IL6) *GeneID:16193*	Forward: 5′-CTCTGGGAAATCGTGGAAAT-3′ Reverse: 5′-CCAGTTTGGTAGCATCCATC-3′
Lipoprotein lipase (LPL) *GeneID: 16956*	Forward: 5′-GGGCCCAGCAACATTATCCA-3′ Reverse: 5′-GGGGGCTTCTGCATACTCAA-3′
Major histocompatibility complex class II (MHC II) *GeneID: 14961*	Forward: 5′-CAGACGCCGAGTACTGGAAC-3′ Reverse: 5′-CAGCGCACTTTGATCTTGGC-3′
Neprilysin *GeneID: 17380*	Forward: 5′-GAGAAAAGCCCACTTGCTTG-3′ Reverse: 5′-GAAAGACAAAATGGGGCAGA-3′
Phosphoglycerate kinase 1 (Pgk1) *GeneID: 18655*	Forward: 5′-GCCTGTTGACTTTGTCACTGC-3′ Reverse: 5′-GAGTGACTTGGTTCCCCTGG-3′
Succinate dehydrogenase complex, subunit A, flavoprotein (SDHA) *Gene ID: 66945*	Forward: 5′-ACACAGACCTGGTGGAGACC-3′ Reverse: 5′-GGATGGGCTTGGAGTAATCA-3′
Tumor necrosis factor α (TNFα), transcript variant 1 *GeneID: 21926*	Forward: 5′-CCCTCACACTCAGATCATCTTCT-3′ Reverse: 5′-GCTACGACGTGGGCTACAG-5′

### β-Amyloid enzyme-linked immunosorbent assay

Levels of soluble Aβ42 peptides were determined by enzyme-linked immunosorbent assay (ELISA) as described previously [69], with noted modifications. Briefly, the remaining hemi-brain portions were homogenized in buffer (0.2% diethylamine, 50 mM NaCl, 1 mL/200 mg tissue) using a polytron on ice. Resulting homogenates were centrifuged at 4 °C for 1 h at 15,000 g. Supernatants were collected and neutralized with 1/10th volume of 0.5 M Tris-HCl, pH 6.8. Samples were then analyzed using a commercially available Aβ42 ELISA (Human/Rat β Amyloid 42 ELISA Kit High Sensitive; 292-64501; Wako Chemicals) according to manufacturer’s directions.

### Statistical analyses

All data were analyzed using Prism software (version 7, GraphPad Software, La Jolla, CA, USA). Two-way repeated measures ANOVAs were performed for the analyses of body weight and glucose tolerance. All other data were analyzed by two-way ANOVAs. In the case of significant main effects, planned comparisons between groups were made using the Bonferroni correction. All data are represented as the mean ± the standard error of the mean (SEM). Significance was set at a threshold of *p* < 0.05.

## Results

### Effects of HFD and TAK-242 on body weight and adiposity

**Fig. 1:**
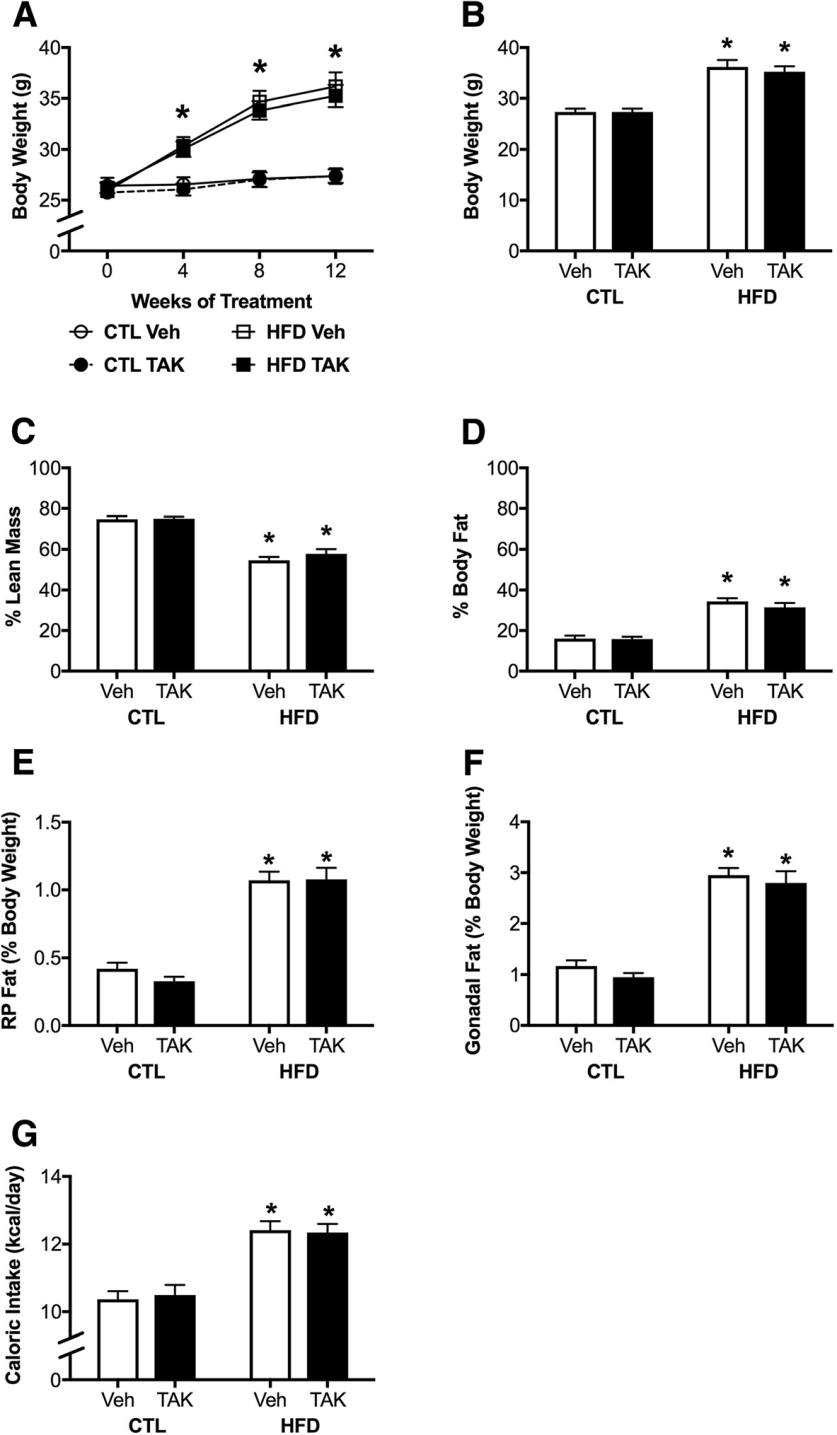
Metabolic outcomes associated with diet-induced obesity in mice treated with vehicle (Veh) or the TLR4 inhibitor TAK-242 (TAK). **a** Body weights in male C57BL6/J mice maintained on control (CTL) and high-fat (HFD) diets and vehicle (Veh) and TAK-242 (TAK) treatments taken at baseline (week 0) and 4-week intervals across the 12-week experimental period, and **b** body weights at the end of the treatment period. **c** Lean mass and **d** percent body fat via NMR scan. **e**, **f** Adiposity as measured by **e** retroperitoneal (RP) fat pad and **f** gonadal fat pad weights. **g** Average daily caloric intake across the experimental period. Data are presented as mean (±SEM) values; *n* = 10–14/group. For data presented across time, control diet-fed mice are shown as circles, high-fat diet-fed mice are shown as squares; vehicle-treated are open symbols, TAK-242-treated are filled symbols; for all other panels, vehicle-treated animals are shown in white bars and TAK-242-treated are shown in black bars. Statistical significance is based on ANOVA followed by Bonferroni correction. * *p* < 0.05 relative to drug treatment-matched mice in control diet condition

We first examined measures of DIO in vehicle-treated and TAK-242-treated animals to assess whether drug treatment altered the obesogenic effects of HFD. The control diet was associated with a ~ 5% weight gain in both vehicle-treated and TAK-242-treated mice, whereas HFD was associated with a 39.6 ± 4.2% increase in body weight in vehicle-treated mice and a 34.4 ± 3.0% increase in TAK-242-treated mice (Fig. 1a). Two-way repeated measures ANOVA showed that HFD significantly increased body weight (*F* = 38.9, *p* < 0.0001). There was no significant effect of drug treatment on body weight. Between-group comparisons revealed that mice fed HFD weighed more than those fed CTL diets at the 4-, 8-, and 12-week time points (*p* < 0.05); this was true for both vehicle-treated and TAK-242-treated groups. When examining final body weight, we found a main effect of diet (*F* = 63.88, *p* < 0.0001; Fig. 1b), which was significant across both drug treatments (*p* < 0.0001). There were no significant interactions between diet and drug on measures of body weight.

Next, we examined adiposity by both body composition analysis and weights of gonadal and RP fat pads. We found that HFD was associated with a significant decrease in percent lean body mass (*F* = 103.0, *p* < 0.0001; Fig. 1c) and a corresponding significant increase in percent body fat (*F* = 98.7, *p* < 0.0001; Fig. 1d) in both vehicle-treated and TAK-242-treated mice (*p* < 0.0001). There was no interaction effect between diet and drug, nor were there significant main effects of drug on lean mass or body fat. The same pattern was found with fat depot weight, such that HFD significantly increased weights of both RP (*F* = 117.1, *p* < 0.0001; Fig. 1e) and gonadal (*F* = 108.4, *p* < 0.0001; Fig. 1f) fat pads. There were neither significant main effects of drug nor interaction effects between diet and drug.

We also examined food intake and found that HFD feeding was associated with a significant increase in the average daily kilocalorie consumption (*F* = 52.5, *p* < 0.0001; Fig. 1g), as would be expected given the higher caloric density of HFD relative to control diet. Importantly, drug treatment did not significantly affect caloric intake, and there were no significant interactions between diet and drug.

### Effects of HFD and TAK-242 on metabolic outcomes

**Fig. 2:**
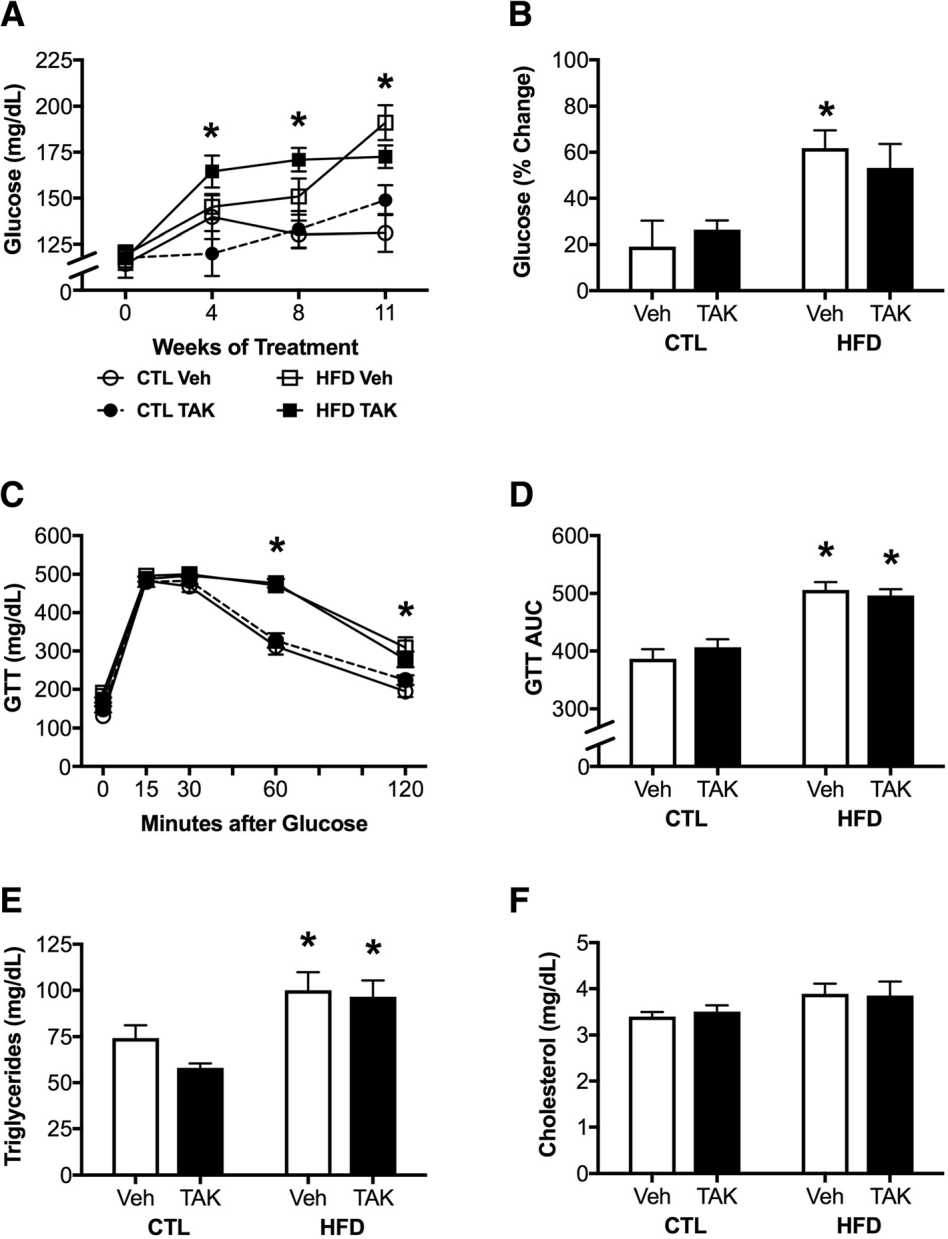
Peripheral effects of diet-induced obesity in mice treated with vehicle (Veh) or the TLR4 inhibitor, TAK-242 (TAK). **a** Baseline fasting glucose levels in male C57BL6/J mice maintained on control (CTL) and high-fat diets (HFD) and drug treatments taken at baseline (week 0) and weeks 4, 8, and 11. **b** Percent change in fasting blood glucose levels relative to baseline after 12 weeks of control or high-fat diet. **c** Glucose tolerance test showing blood glucose levels over time after administration of a glucose bolus and **d** area under the curve (AUC) for the glucose tolerance test. **e** Plasma triglyceride levels and **f** plasma cholesterol levels at the end of the experimental period. Data are presented as mean (±SEM) values; *n* = 10–14/group. For data presented across time, control diet-fed mice are shown as circles, high-fat diet-fed mice are shown as squares; vehicle-treated are open symbols, TAK-242-treated are filled symbols; for all other panels, vehicle-treated animals are shown in white bars and TAK-242-treated are shown in black bars. Statistical significance is based on ANOVA followed by Bonferroni correction. * *p* < 0.05 relative to drug treatment-matched mice in control diet condition

Another established outcome of DIO is dysregulation of glucose homeostasis [70, 71]. We first examined changes in fasting glucose levels over the treatment period. We found that HFD was associated with a significant increase in glucose levels (*F* = 23.78, *p* < 0.0001; Fig. 2a) at the 4-, 8-, and 11-week time points (*p* < 0.05). Additionally, there was a significant main effect of diet on percent change in glucose levels from baseline to the end of the treatment period (*F* = 13.7, *p* < 0.001; Fig. 2b). However, between-group comparisons revealed that the effect of HFD on increasing fasting glucose was only significant in vehicle-treated (*p* < 0.01), not in TAK-242-treated animals. There were neither significant interaction effects nor main effects of drug treatment on these measures of glucose homeostasis.

In addition to fasting glucose levels, we examined responses to a glucose bolus. There was a main effect of diet on glucose clearance in GTT (*F* = 49.95, *p* < 0.0001; Fig. 2c) that was significant in both vehicle-treated and TAK-242-treated mice (*p* < 0.05). We also calculated the AUC for GTT and again found a significant effect of diet (*F* = 55.11, *p* < 0.0001; Fig. 2d) such that HFD increased AUC regardless of drug treatment. There were no significant interactions or main effects of drug treatment on GTT measures.

Finally, we examined the effects of diet and drug treatments on levels of plasma triglycerides and cholesterol. HFD was associated with significantly increased triglyceride levels (*F* = 15.64, *p* < 0.001; Fig. 2e) in both drug treatment groups (*p* < 0.05). There was a non-significant trend towards increased cholesterol levels by HFD (*F* = 3.41, *p* = 0.07; Fig. 2f). There were no main effects of drug nor were there interactions between diet and drug on levels of either triglycerides or cholesterol.

### Effects of HFD and TAK-242 on peripheral inflammation

**Fig. 3:**
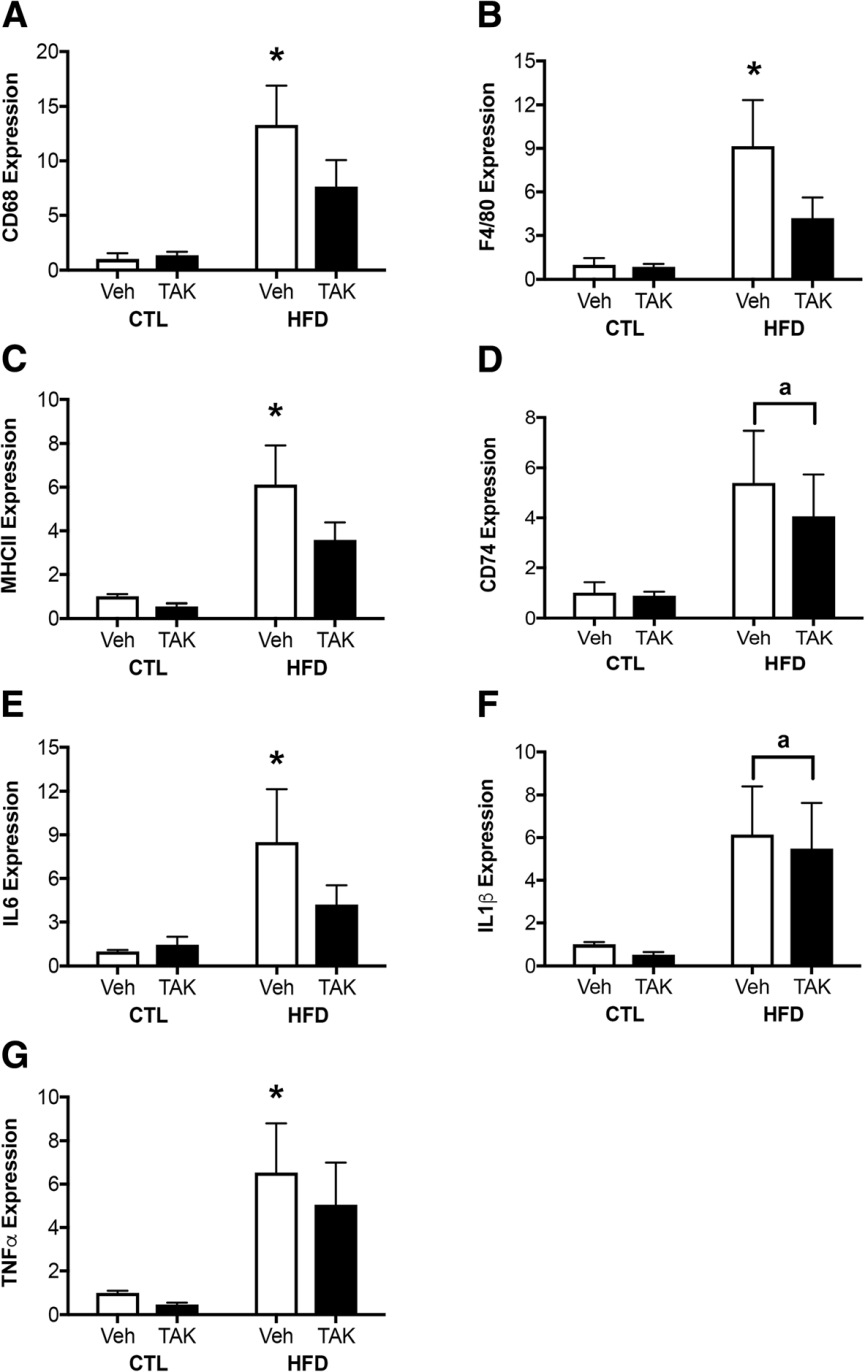
Expression of mRNA levels of genes associated with macrophage activation and inflammation in adipose tissue from vehicle (Veh) and TAK-242 (TAK)-treated mice fed a control (CTL) or high-fat diet (HFD). mRNA from gonadal fat pads was probed for levels of the macrophage markers **a** CD68 and **b** F4/80, as well as for the antigen presenting molecules **c** MHC II and **d** CD74. Pro-inflammatory cytokine expression was examined by probing for levels of **e** IL-6, **f** IL-1β, and **g** TNFα. Data show fold difference means and standard error of the means (SEM) relative to vehicle-treated mice fed a control diet *n* = 10/group. Vehicle-treated animals are shown in white bars and TAK-242-treated are shown in black bars. Statistical significance is based on ANOVA followed by Bonferroni correction. *p < 0.05 relative to drug treatment-matched mice in control diet condition. ^a^*p* < 0.05 for main effect of diet that does not reach statistical significance in between-group comparisons

DIO is known to increase inflammation in a number of organs, including adipose tissue [72]. To assess effects of HFD on peripheral tissue inflammation, we examined gene expression of markers of macrophage activation and inflammatory cytokines in gonadal fat (Fig. 3). We found a significant effect of diet on adipose tissue levels of CD68 (*F* = 17.14, *p* < 0.001; Fig. 3a), F4/80 (*F* = 10.06, *p* < 0.01; Fig. 3b), MHCII (*F* = 16.42, *p* < 0.001; Fig. 3c), CD74 (*F* = 7.42, *p* < 0.05; Fig. 3d), IL-6 (*F* = 6.52, *p* < 0.05; Fig. 3e), IL-1β (*F* = 9.99, *p* < 0.01; Fig. 3f), and TNFα (*F* = 10.85, *p* < 0.01; Fig. 3g). However, for the markers of CD68, F4/80, MHC II, IL6, and TNFα, this effect was only statistically significant in vehicle-treated HFD-fed mice and did not reach statistical significance in TAK-242-treated HFD-fed mice. For CD74 and IL-1β the main effect of diet failed to reach statistical significance in either of the HFD-fed groups. There was neither a main effect of drug, nor an interaction effect between diet and drug on expression of any probed genes.

### Effects of HFD and TAK-242 on hippocampal microgliosis

**Fig. 4:**
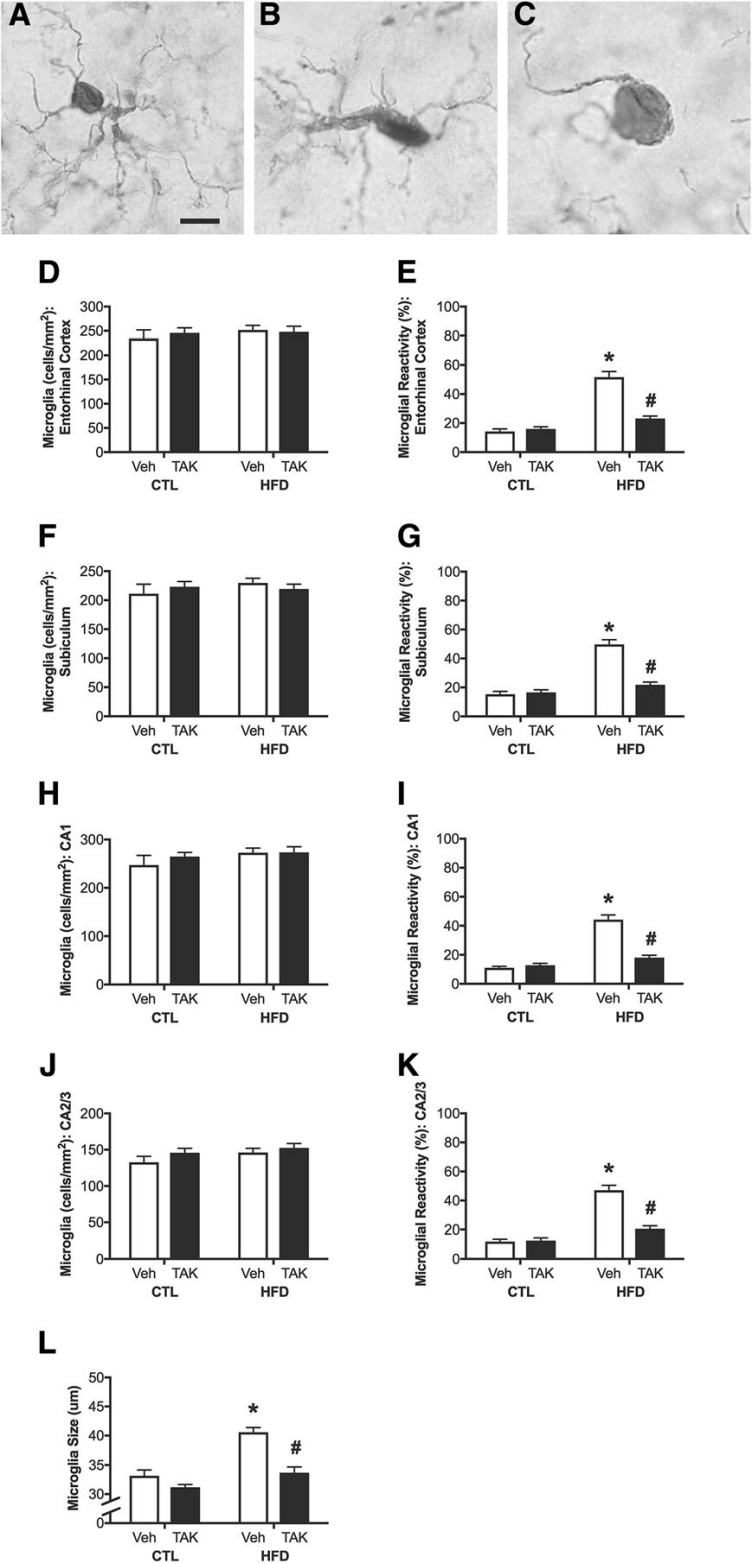
Microglial number, morphological status, and soma size as assessed by IBA-1 immunohistochemistry in control (CTL) and high-fat diet (HFD)-fed mice treated with vehicle (Veh) or the TLR4 inhibitor, TAK-242 (TAK). **a**–**c** Representative images of microglial morphology. Scale bar = 10 μm. **a** Resting (type 1) microglial cells are characterized by small cell bodies with numerous branching processes. Reactive microglia are either **b** type 2 cells with rod-shaped cell bodies and fewer, thicker projections, or **c** amoeboid cells with no processes or with filopodia. Densities of IBA-1 immunoreactive cells were quantified in **d** entorhinal cortex and in the **f** subiculum, **h** CA1, and **j** CA2/3 of the hippocampus. Percentages of reactive microglia (type 2 and 3 cells) were quantified in **e** entorhinal cortex and in the hippocampal subregions **g** subiculum, **i** CA1, and **k** CA2/3. **l** Microglial soma size was assessed specifically in CA1 of the hippocampus. Data are presented as mean (±SEM) values; *n* = 10/group. Vehicle-treated animals are shown in white bars, and TAK-242-treated are shown in black bars. Statistical significance is based on ANOVA followed by Bonferroni correction. *p < 0.05 relative to drug treatment-matched mice in control diet condition. #*p* < 0.05 relative to vehicle-treated mice in the same diet condition

In addition to causing macrophage activation and inflammation in peripheral tissues, HFD is associated with increased glial activation and inflammation in the brain [73, 74]. Because microglia express high levels of TLR4 [57, 58] and have been shown to adopt activated phenotypes in response to HFD [40, 75, 76], we examined microgliosis in brain sections. We analyzed both cell density and morphology of labeled cells following immunostaining for IBA-1 in entorhinal cortex and in the subiculum, CA1, and CA2/3 regions of the hippocampus. Figure 4a–c illustrates morphological phenotype characteristic of resting (type 1; Fig. 4a) microglia, with multiple, thin processes, and activated microglia with fewer, thicker processes (type 2; Fig. 4b), or amoeboid appearance (type 3; Fig. 4c). When examining microglial density, we found neither significant effects of diet or drug treatment, nor an interaction between these factors in entorhinal cortex (Fig. 4d), subiculum (Fig. 4f), CA1 (Fig. 4h), or CA2/3 (Fig. 4j). However, we found significant interactions between diet and drug treatment on microglial activation in entorhinal cortex (*F* = 36.27, *p* < 0.0001; Fig. 4e), subiculum (*F* = 38.93, *p* < 0.0001; Fig. 4g), CA1 (*F* = 47.16, *p* < 0.0001; Fig. 4i), and CA2/3 (*F* = 31.7, *p* < 0.0001; Fig. 4k). Between-group comparisons revealed that across all brain regions, HFD increased microglial reactivity exclusively in vehicle-treated animals, and TAK-242 was associated with significantly reduced microglial reactivity specifically in HFD-fed animals.

Activation phenotypes of microglia are also associated with increased soma size [65]. We measured microglial soma size as a complementary measure of microgliosis, specifically in the CA1 region of the hippocampus. Our data show similar results to findings on microglial morphology. That is, there is a significant interaction effect between diet and drug treatment (*F* = 8.62, *p* < 0.01; Fig. 4l), such that diet significantly increased soma size only in vehicle-treated animals (*p* < 0.0001), and TAK-242 treatment was associated with significantly decreased soma size compared to the vehicle group only in HFD-fed animals (*p* < 0.0001).

### Effects of HFD and TAK-242 on hippocampal gene expression

**Fig. 5:**
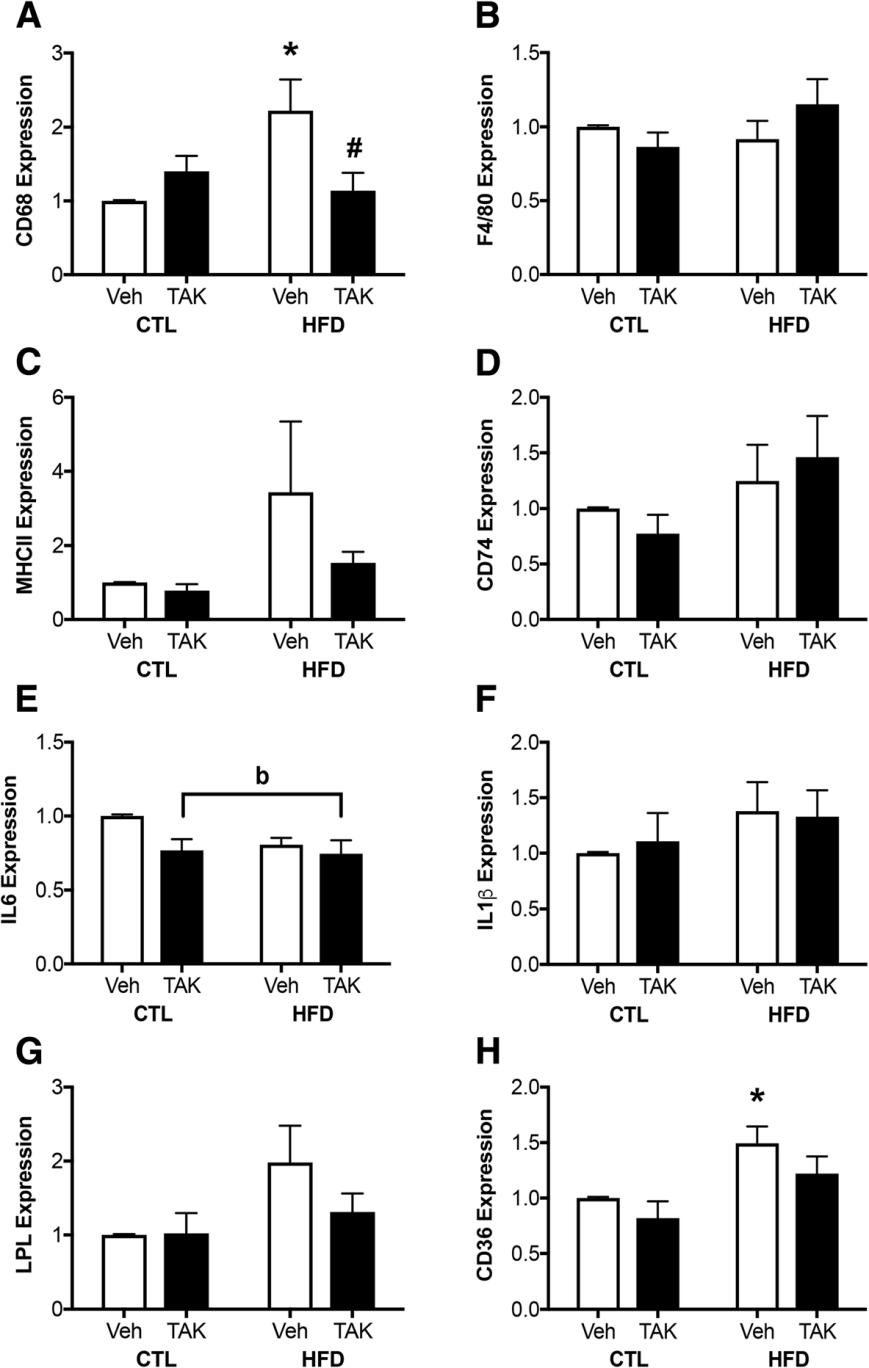
Hippocampal mRNA expression of genes associated with activated microglial phenotypes and neuroinflammation in mice fed a control (CTL) or high-fat diet (HFD) and treated with vehicle (Veh) or the TLR4 inhibitor, TAK-242 (TAK). Hippocampal mRNA was probed for levels of the microglia/macrophage markers **a** CD68 and **b** F4/80, and for the innate immune antigen presentation markers **c** MHC II and **d** CD74. Pro-inflammatory cytokine expression was examined using **e** IL-6 and **f** IL-1β. Gene expression of two factors involved in microglial phenotype, lipid transport, and uptake **g** LPL and **h** CD36 were also assayed in hippocampus. Data show fold difference means and standard error of the means (SEM) relative to vehicle-treated mice fed a control diet *n* = 10/group. Vehicle-treated animals are shown in white bars and TAK-242-treated are shown in black bars. Statistical significance is based on ANOVA followed by Bonferroni correction. *p < 0.05 relative to drug treatment-matched mice in control diet condition. #*p* < 0.05 relative to vehicle-treated mice in same diet condition. ^b^*p* < 0.05 for main effect of drug treatment that does not reach statistical significance in between-group comparisons

One frequent consequence of microgliosis is the increased expression of various microglia/macrophage markers and pro-inflammatory cytokines [77, 78]. We examined gene expression levels of several such factors in hippocampus (Fig. 5), including CD68 and F4/80 as general microglia/macrophage markers [79], with CD68 being characteristic of a more activated cell phenotype [80–82]. We found a significant interaction between diet and drug (*F* = 7.06, *p* < 0.05; Fig. 5a) on expression levels of CD68. Between-group comparisons revealed that HFD increased CD68 expression only in vehicle-treated but not in TAK-242-treated animals (*p* < 0.01), and TAK-242 significantly decreased CD68 only in HFD-fed mice (*p* < 0.05). There were neither significant effects of diet or drug treatment, nor an interaction between these factors on hippocampal gene expression of F4/80 (Fig. 5b).

We next examined levels of MHC II, which is expressed specifically in microglia in the brain [83, 84], enhances TLR4 signaling [85], and is increased in microglia by HFD [10], and of CD74, which is also expressed specifically by microglia and macrophages [86], and is involved in the formation and transport of MHC II [87]. Additionally, CD74 expression has been shown to correlate with obesity-induced increases in body weight and metabolic changes in adipose tissue [88], and is increased in hippocampus of HFD-fed mice [89]. There was a statistically non-significant trend of increased MHC II in the HFD-vehicle group (Fig. 5c), and no significant diet or drug effects on CD74 expression (Fig. 5d).

Increased production of pro-inflammatory cytokines is a one potential outcome of increased microglial activation [78] as well as of obesity [21]. Thus, we probed for two pro-inflammatory cytokines: IL-6 (Fig. 5e) and IL-1β (Fig. 5f). Though diet did not significantly affect gene expression of either cytokine, we found a significant main effect of drug on levels of IL-6 (*F* = 4.73, *p* < 0.05); however, this did not reach statistical significance in either CTL-fed or HFD-fed mice.

Finally, we examined mRNA levels of two factors involved in fatty acid transport and uptake as well as regulation of activated microglial phenotypes: LPL [90, 91] and CD36 [92, 93]. Results demonstrated a statistically non-significant trend of a main effect of diet on LPL levels (*F* = 3.03, *p* = 0.08; Fig. 5g), as well as a statistically significant effect of diet on CD36 expression (*F* = 10.99; *p* < 0.01; Fig. 5h). Between-group comparisons showed that HFD significantly increased CD36 only in vehicle-treated but not in TAK-242-treated mice (*p* < 0.05).

### Effects of HFD and TAK-242 on neurogenesis

**Fig. 6:**
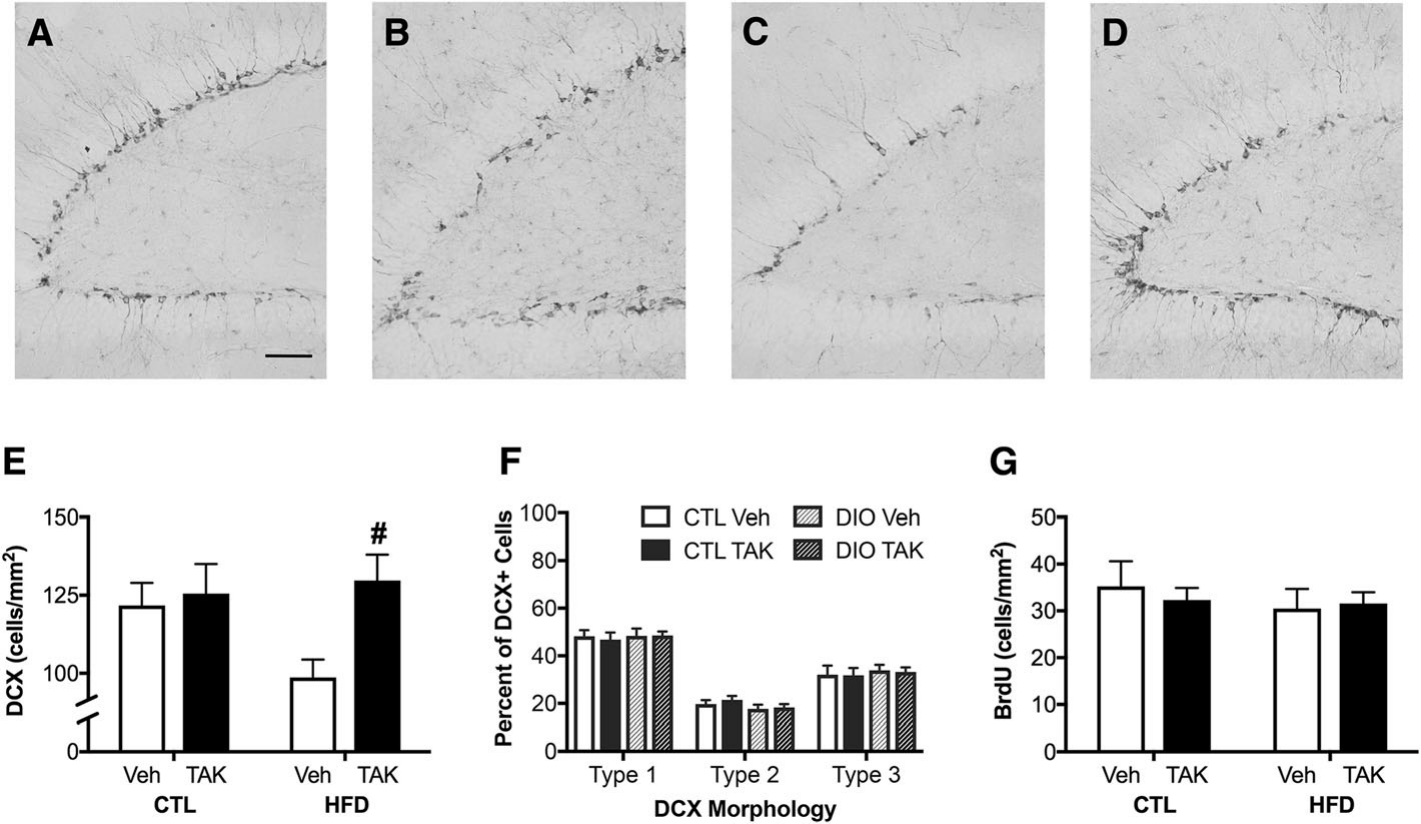
Neurogenesis and cell proliferation as assessed by DCX and BrdU immunohistochemistry in mice maintained on control (CTL) or high-fat diets (HFD) and treated with vehicle (Veh) or the TLR4 inhibitor, TAK-242 (TAK). **a**–**d** Representative images of DCX immunohistochemistry in mice treated with **a** control diet (CTL) and vehicle (Veh), **b** control diet and TAK-242 (TAK), **c** high-fat diet (HFD) and vehicle, and **d** high-fat diet and TAK-242. Scale bar = 50 μm. **e** Densities of DCX immunoreactive cells were quantified in the dentate gyrus. **f** The maturation state of DCX-positive cells was assessed, and cells were categorized as type 1, type 2, or type 3 based on their dendritic morphology. **g** BrdU-positive cells were quantified in the dentate gyrus. Data are presented as mean (±SEM) values; *n* = 10/group. Vehicle-treated animals are shown in white bars, and TAK-242-treated are shown in black bars; for DCX morphology, CTL diet-fed animals are shown in solid bars and HFD-fed animals are shown in striped bars. Statistical significance is based on ANOVA followed by Bonferroni correction. #*p* < 0.05 relative to vehicle-treated mice in the same diet condition

An established negative consequence of DIO is impaired neurogenesis [8, 9]. We examined neurogenesis across groups using techniques to quantify both neural stem cells committed to a neuronal phenotype (DCX-labeling) and proliferation of neural stem cells (BrdU labeling) in the dentate gyrus region of the hippocampus. Figure 6 shows representative images of DCX immunohistochemistry, which qualitatively show a decrease in labeled cells with HFD that is prevented by TAK-242 treatment (Fig. 6a–d). Quantification of DCX-labeled cell density showed a significant main effect of drug (*F* = 5.01, *p* < 0.05; Fig. 6e). Between-group comparisons revealed that this effect of drug treatment was significant only in HFD-fed mice (*p* < 0.05). In addition, there was a non-significant trend towards an interaction between diet and drug (*F* = 3.05, *p* = 0.08; Fig. 6e). There was no significant effect of diet on DCX-labeled cells. We also determined the relative maturity of DCX-positive cells by examining their dendritic morphology and arborization [66, 67]. Diet and drug treatments did not significantly affect maturation states of new neurons, as the proportion of subtypes was roughly equivalent between treatment groups (Fig. 6f). Parallel assessment of BrdU-labeled cells revealed neither significant main effects of diet or drug, nor an interaction between these factors (Fig. 6g).

### Effects of HFD and TAK-242 on amyloidogenic pathways

**Fig. 7:**
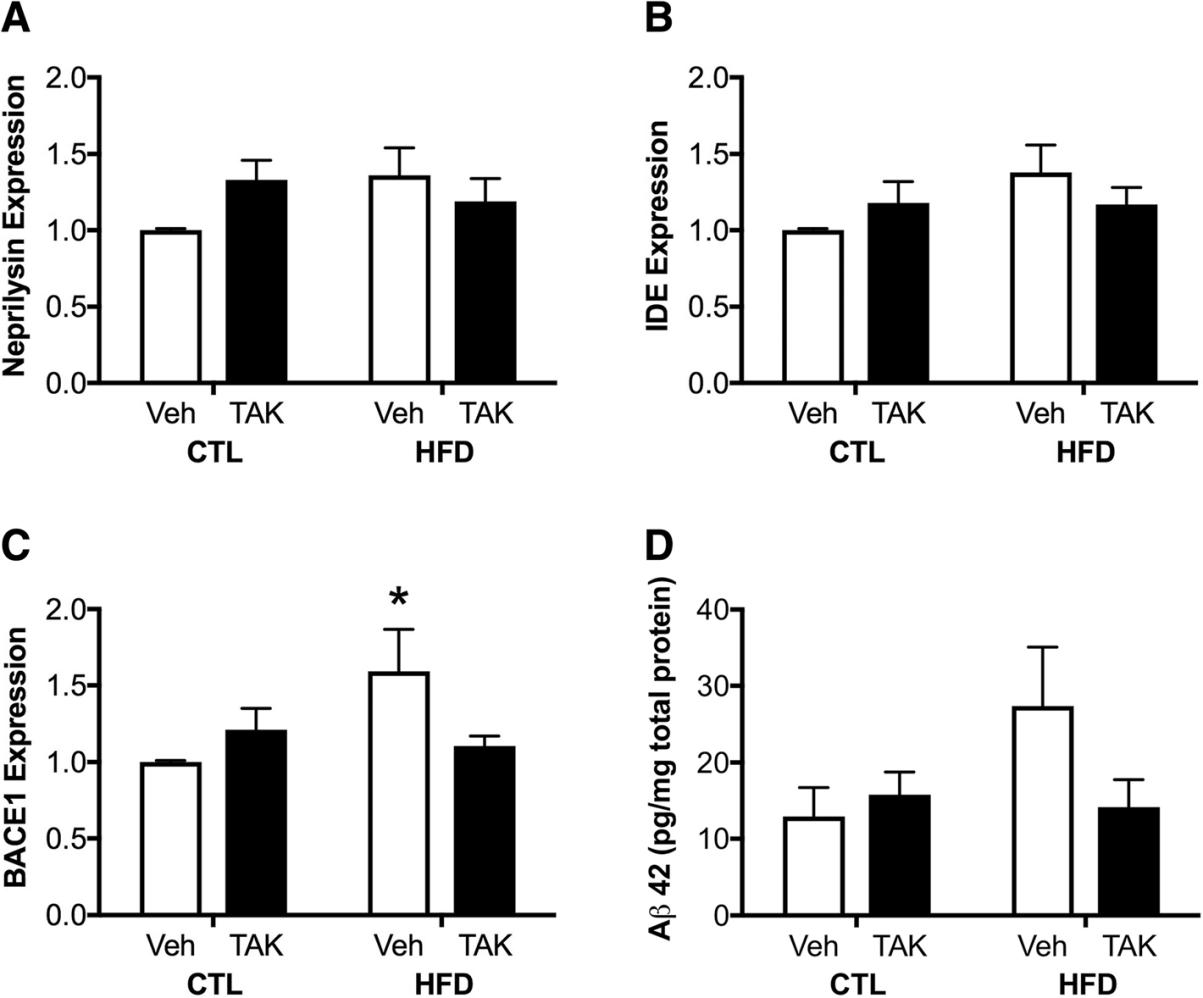
Expression of Aβ production and degrading factors and soluble Aβ42 in mice fed a control (CTL) or high-fat diet (HFD) and treated with vehicle (Veh) or the TLR4 inhibitor TAK-242 (TAK). **a**–**c** Hippocampal gene expression of the Aβ clearance factors **a** neprilysin, and **b** insulin-degrading enzyme, and the Aβ production factor **c** BACE1, as assessed by qPCR. Data show fold differences relative to vehicle-treated mice fed a control diet. **d** Protein levels of soluble Aβ42 as measured by ELISA (expressed as pg Aβ42 per to mg total protein). Data are presented as mean (±SEM) values; *n* = 10/group. Vehicle-treated animals are shown in white bars, and TAK-242-treated are shown in black bars. Statistical significance is based on ANOVA followed by Bonferroni correction. *p < 0.05 relative to drug treatment-matched mice in control diet condition

DIO has been shown to promote Alzheimer-related amyloidogenic pathways in rodent models, in part by regulating neural expression of factors involved in Aβ production and clearance [94–98]. We measured hippocampal expression levels of three key genes to investigate if HFD and TAK-242 treatments affect Aβ homeostasis pathways. We found no significant effects of diet or drug treatment, nor an interaction between these factors, on expression levels of the Aβ-degrading enzymes neprilysin (Fig. 7a) and IDE (Fig. 7b). However, we found a significant interaction between diet and drug treatment (*F* = 4.90, *p* < 0.05; Fig. 7c) on levels of the pro-amyloidogenic Aβ enzyme BACE1. Between-group comparisons revealed that HFD significantly increased BACE1 in vehicle-treated animals (*p* < 0.05) but not in TAK-242-treated mice. Finally, we determined levels of soluble Aβ42 peptides by ELISA. There were no statistically significant effects of diet or drug treatment and no interaction between these factors, though there were non-significant trends consistent with the BACE1 data (Fig. 7d).

### Effects of HFD and TAK-242 on exploration, anxiety-like, and depressive-like behaviors

To determine whether the drug treatment affected general behavioral performance, we compared animals on measures of activity, anxiety, and depression, using the behavioral assays of open field, elevated plus maze, and forced swim test, respectively. We found no statistically significant main effects of drug treatment on any of these three behavioral tasks (Additional file 1: Figure S1). There were only two statistically significant effects on any of the outcome measures in these assays, and both of these were in exploratory activity in the open field. First, there was a significant interaction between diet and drug treatment on the number of times the animals crossed into the center field (*F* = 4.91, *p* < 0.05; Additional file 1: Figure S1A), such that HFD increased center crossings only in TAK-242-treated mice (*p* < 0.05). Second, there was a significant main effect of diet on time spent in the center field (*F* = 4.23, *p* < 0.05; Additional file 1: Figure S1B), such that HFD again increased this measure specifically in TAK-242-treated animals (*p* < 0.05). There were no statistically significant effects on any measures of anxiety-like or depressive-like behaviors.

### Effects of HFD and TAK-242 on behavioral performance in cognitive tasks

**Fig. 8:**
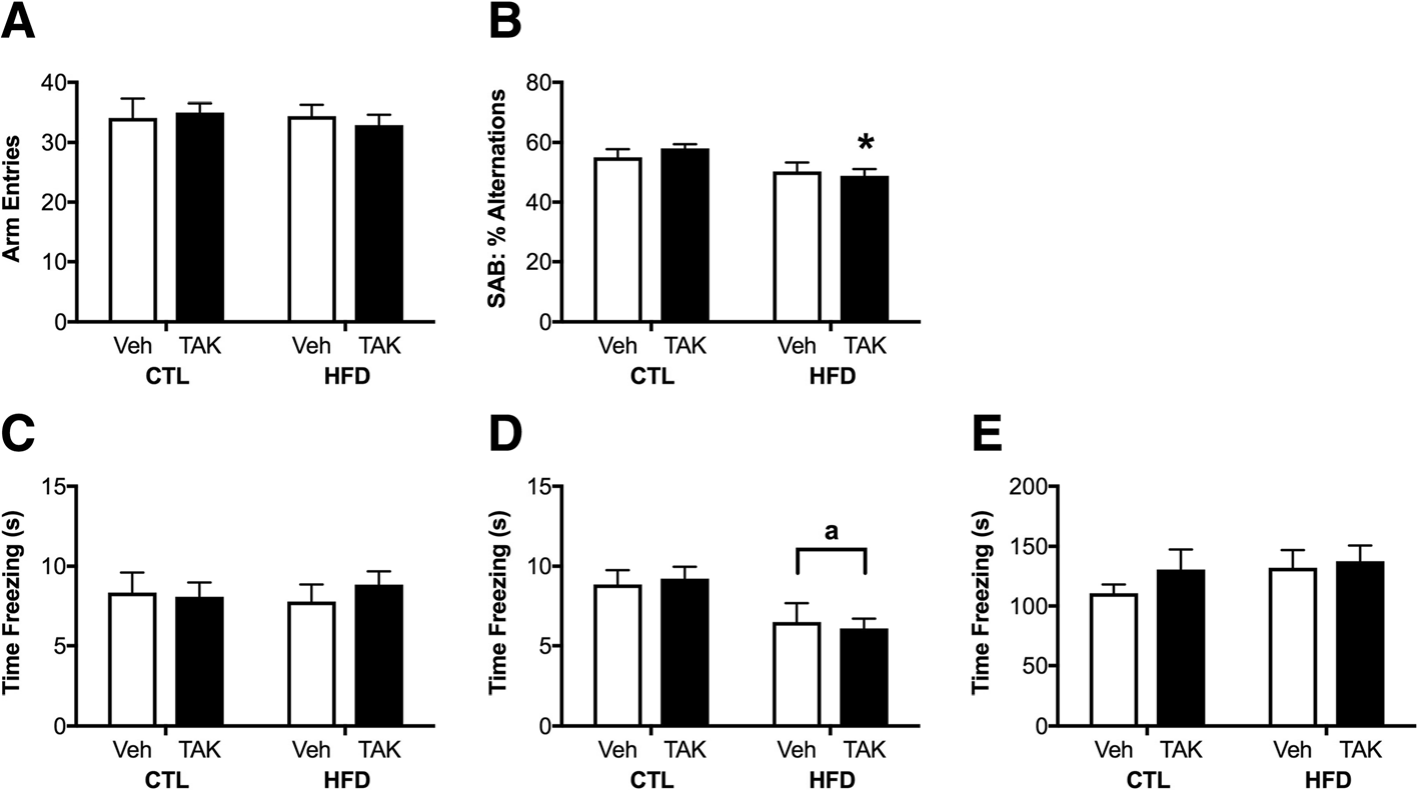
Working memory and cued and contextual memory performance in mice maintained on control (CTL) or high-fat diet (HFD) and given vehicle (Veh) or the TLR4 inhibitor, TAK-242 (TAK). **a**, **b** Short-term working memory was assessed by spontaneous alternation behavior. **a** Total arm entries in the Y maze. **b** Spontaneous alternation behavior (SAB) in the Y maze. **c**–**e** Cued and contextual memory was tested in the fear conditioning paradigm. **c** Learning was assessed by examining freezing behavior during the trace period between the tone and shock on the 5th trial of the training day. **d** Cued memory was tested 24 h later in a different context. **e** Contextual memory was assessed 24 h after the cued test. Data are presented as mean (±SEM) values; *n* = 10–14/group. Vehicle-treated animals are shown in white bars, and TAK-242-treated are shown in black bars. Statistical significance is based on ANOVA followed by Bonferroni correction. *p < 0.05 relative to drug treatment-matched mice in control diet condition

Finally, we examined cognitive performance in two behavioral assays. The spontaneous alternation behavior (SAB) task assays short-term working memory and visual attention. Total arm entries did not vary by either diet or drug treatment (Fig. 8a). However, there was a main effect of diet on alternation behavior (*F* = 7.86, *p* < 0.01; Fig. 8b), which was significantly only in TAK-242-treated mice (*p* < 0.05), though performance of the TAK-242-treated HFD mice (49%) is not significantly different from that observed in vehicle-treated HFD mice (50%).

As a measure of hippocampal-dependent and hippocampal-independent memory, we tested animals using the fear-conditioning paradigm. When examining freezing during the day 1 training trials, we found no significant differences between groups in initial freezing before the tone/shock pairing or in freezing during the tone/shock pairings and inter-trial periods. Figure 8c shows time spent freezing during the trace period between the tone and shock during the final presentation of the tone/shock pairing on day 1.

Cued memory was assessed on day 2 by changing the appearance and odor of the chamber and examining freezing to the tone, without presenting the shock. There were no group differences in freezing during the baseline period before presentation of the tone (data not shown). There was a main effect of diet on freezing during the trace period immediately after the first presentation of the tone (*F* = 4.76, *p* < 0.05; Fig. 8d), but this did not reach statistical significance across the different drug treatments. There were no significant group differences on freezing during the following 2 tone presentations (data not shown).

Contextual memory was examined on day 3 by placing animals back into the chamber with the same appearance and odor as during day 1 and examining freezing to this context. There were no significant effects of either diet or drug treatment, nor was there an interaction between these factors, on freezing in response to the context (Fig. 8e).

## Discussion

The goal of this study is to examine the role of TLR4 signaling in mediating the effects of obesity on microglial activation and adverse neural outcomes. Comparing animals fed control versus HFD in the presence or absence of the TLR4 inhibitor TAK-242, we demonstrate that TAK-242 treatment was associated with attenuation of HFD-induced adipose tissue inflammation, microgliosis, and reduction in neurogenesis in the hippocampus. However, TAK-242 treatment did not improve the metabolic dysregulation induced by HFD feeding. The finding that TLR4 inhibition did not protect against effects of HFD on weight gain and adiposity is consistent with numerous other studies [42, 44–46, 49, 52, 53]. In contrast to our findings, however, many of these studies show that obesity-associated dysregulation of insulin and glucose signaling was improved in the absence of TLR4 signaling [42–47, 53]. One possible reason for this discordance is that several studies used mice with either knockout or dysfunctional TLR4, whereas we used a pharmacological approach to inhibit TLR4. Constitutive absence of TLR4 signaling may result in metabolic changes even in the absence of HFD and is likely to result in more complete inhibition of TLR4 and different outcomes than pharmacological approaches.

Our findings support the conclusion that TLR4 contributes to obesity-induced activation of peripheral macrophages and brain microglia. First, our observations in adipose tissue of partial reductions in both markers of macrophage activation (CD68, F4/80, MHCII) and pro-inflammatory cytokines (IL-6, TNFα) in HFD-fed mice treated with TAK-242 is consistent with previous findings [42, 44, 46, 48, 53]. Second, we demonstrate that the TLR4 inhibitor attenuates HFD-induced microgliosis in hippocampus, as evidenced by changes in microglial morphology and soma size and mRNA levels of the activated microglia markers CD68 and, to a lesser extent, CD36 or fatty acid translocase. CD36 is a pattern recognition receptor that exhibits increased expression in obesity [93] as well as in AD [99, 100], where it mediates recruitment of microglia to Aβ deposits [101, 102]. Interestingly, CD36 has been found to form a complex with TLR4 and TLR6 through which both Aβ and lipids can induce inflammation [103]. Collectively, these findings support and extend prior work by Milanski and colleagues that implicated TLR4 in obesity-induced glial activation in hypothalamus, which may contribute to systemic metabolic disturbances [54, 55].

Although we observed an increase in activated microglia in response to HFD, it is noteworthy that hippocampal expression of the pro-inflammatory cytokines IL-6 and IL1-β was not increased by obesogenic diet. Though HFD is often associated with both microglial activation and increased cytokine expression [104], others show changes only in some brain regions [105], or no changes in pro-inflammatory cytokines [106]. Here, we find DIO-associated changes in specific markers of activated microglia but not in cytokines, which is consistent with previous work by Setti and colleagues [89]. Although it is reasonable to predict that a more chronic exposure to HFD may be required for increased neural cytokine expression, cytokine expression in hypothalamus is significantly increased by high-fat diet exposures as brief as 1 day [107]. We posit that the observed microglial activation in the absence of significantly increased expression of pro-inflammatory cytokines is consistent with the known heterogeneity in activated microglial phenotypes [29, 30]. Indeed, accumulating evidence indicates that deleterious effects of microglia are mediated by numerous factors rather than simply increased levels of pro-inflammatory cytokines [32, 108]. The extent to which various activated microglial phenotypes differentially affect neural outcomes is an important topic that remains to be fully elucidated.

One deleterious neural consequence common to both diet-induced obesity and activated microglia is promotion of amyloidogenesis. HFD is known to increase gene expression and/or enzyme activity of the pro-amyloidogenic BACE1 [95] and decrease levels of the Aβ-degrading enzymes neprilysin [94] and insulin-degrading enzyme [97]. These effects on Aβ homeostasis likely contribute to observations that experimental obesity drives Aβ accumulation in transgenic mouse models of AD [13, 14]. Additionally, it has been shown that inflammation can increase levels of BACE1 [109] and decrease levels of neprilysin [110]. Thus, microglial activation and associated neuroinflammation are likely significant mediators of the obesity-induced increase in Aβ. We found that HFD significantly increased levels of BACE1 in vehicle-treated, but not in TAK-242-treated mice. This suggests that HFD caused a shift towards more pro-amyloidogenic processing via TLR4 signaling. Although not statistically significant, our analyses of soluble brain Aβ42 showed trends towards increased levels in HFD mice in the absence but not the presence of TLR4 inhibitor.

Another negative effect of obesity is attenuation of neurogenesis. We found that treatment with TAK-242 significantly increased the number of new neurons in dentate gyrus specifically in HFD-fed mice, indicating a protective effect of TLR4 inhibition on obesity-related impairment in neurogenesis. Because BrdU labeling, a marker of cell proliferation, was not affected by diet or drug treatments, the protective effect of TAK-242 appears to involve the survival and or differentiation of newborn neurons rather than stem cell proliferation. The possibility that TLR4 inhibition yielded a generalized increase in new neuron survival is consistent with our finding that the relative proportion of subtypes of newly formed neurons was not significantly altered by either diet or TLR4 inhibition. The reported effects of HFD on neurogenesis and cell proliferation are somewhat mixed in the literature, with some studies finding decreases in both [111, 112] and others finding changes in only one [9, 113] or neither [114] marker of neurogenesis. Differences in experimental parameters including the composition of the diet may affect the extent to which cell proliferation and/or survival of newborn neurons are affected by HFD. Our observed effects were likely mediated by microglia, which have previously been shown to attenuate neurogenesis during states of activation such as after LPS [115, 116] or seizure [26]. Further, our finding of increased neurogenesis with TAK-242 treatment in HFD-fed mice is consistent with prior data showing that TLR4 signaling regulates neurogenesis in response to neural injury and microgliosis [117, 118]. Importantly, adult neurogenesis is regulated both positively and negatively by a range of activated microglial phenotypes [32, 119], reinforcing the emerging complexity of the associations between microglial functions and their activation states.

One limitation of this study is that we were not able to fully determine the effects of TLR4 inhibition on HFD-induced behavioral changes. Although behavioral impairment is often associated with obesity, we found very subtle effects of diet and drug treatments on overall behavioral outcomes. Specifically, mice fed HFD and treated with TAK-242 showed small but significantly increased exploratory behavior/decreased anxiety-like behavior in the open field test, and worse spontaneous alternation in the Y-maze, and HFD was associated with decreased cued memory in fear conditioning. There were no significant effects of our diet or drug manipulations on anxiety-like behavior in EPM, depressive-like behavior in forced swim, or on contextual fear conditioning. Though a number of studies demonstrated cognitive impairments after HFD exposure [120–124], others did not [125–128]. The age at which rodents are exposed to diet-induced obesity may be a factor. For example, one study found significant effects of HFD on behavior in mice started on diet at 5 weeks of age, but not in animals started at 8 weeks [129], whereas another found behavioral impairments in response to HFD in aged but not young adult rats [130]. These studies suggest that the age at which exposure to HFD occurs may be important in determining whether behavioral deficits are observed. As with the induction of neuroinflammation, it is unlikely that the length of HFD exposure is the key variable in whether or not behavioral impairment occurs. Previous studies of HFD outcomes in rodents have showed changes in both neuroinflammation [107, 130] and behavioral outcomes [130, 131] within 3 days of HFD feeding. Moreover, deficits in cognitive performance have also been observed after 9 days [132], 1 month [133], and 3 months [131] of diet exposure.

Though complete elucidation of the mechanisms underlying the effects of obesity on the brain remains to be established, our findings suggest a stronger role for microglial activation than for metabolic dysregulation. That is, despite having similar weight gain and metabolic outcomes in response to HFD, mice treated with a TLR4 inhibitor showed significant reductions in microglial activation and increased neurogenesis in comparison to vehicle-treated mice. This position is consistent with findings in the human literature that the effects of obesity on cognitive impairment are mediated largely by glial/inflammatory rather than metabolic factors [134–136]. Because TAK-242 has systemic effects and we observed partial attenuation of inflammation in adipose tissue, peripheral effects of TLR4 inhibition may have contributed to the observed neural benefits. The role of other mechanisms like vascular and microbiota changes in the effects of obesity on the brain cannot be ruled out and should be addressed in future studies, especially given that inflammation may be important in these systems as well [137, 138].

## Conclusions

To our knowledge, this study provides the first evidence that TLR4 signaling significantly contributes to the adverse effects of obesity on the hippocampus. Though TLR4 is well established as mediating the effects of saturated fatty acids on adverse outcomes in metabolic measures [42–47, 53] and inflammation [42, 44–48, 50, 53], its regulation of obesity-related changes in brain have not been thoroughly investigated. Our data demonstrate that treatment with an inhibitor of TLR4 signaling in the context of obesogenic diet attenuates microgliosis, increases neurogenesis, and trends towards reductions in pro-amyloidogenic pathways. These findings implicate a significant role for microglial function as a key mediator of the neural effects of obesity. Additionally, these findings point to TLR4 as a therapeutic target for obesity, which has important health implications for a range of systemic and neural disorders including type 2 diabetes, cardiovascular disease, and dementia.

## Additional file

Additional file 1: **Figure S1.** Effects of diet and TLR4 inhibition on exploration, anxiety-like, and depressive-like behaviors. Exploration, anxiety-like, and depressive-like behaviors in control (CTL) and high-fat diet (HFD)-fed mice treated with vehicle (Veh) or the TLR4 inhibitor, TAK-242 (TAK). A-C) Explorative and anxiety-like behaviors were examined in the open field. A) The number of times animals entered the center square of the open field and B) the amount of time they spent in the center field. C) General locomotor activity as assessed by the total number of square crossings. D-F) Anxiety-like behavior was assessed in the elevated plus maze. D) The amount of time spent in the open arm of the maze, and E) the number of times the animals crossed into the open arm. F) The latency to enter the open arm for the first time. G-I) Depressive-like behaviors were examined in the forced swim test. G) The total amount of time the animals spent immobile and H) the number of times the animals were immobile. I) The length of the single longest time spent immobile. Vehicle-treated animals are shown in white bars and TAK-242-treated are shown in black bars. *p < 0.05 relative to drug treatment-matched mice in control diet condition. (TIFF 681 kb)

## Abbreviations

AD: Alzheimer’s disease
ANOVA: Analysis of variance
AUC: Area under the curve
Aβ: β-Amyloid
BACE1: β-Site APP cleaving enzyme
BrdU: Bromodeoxyuridine
CD36: Cluster of differentiation 36
CD68: Cluster of differentiation 68
CD74: Cluster of differentiation 74
CTL: Control
DCX: Doublecortin
DIO: Diet-induced obesity
ELISA: Enzyme-linked immunosorbent assay
EPM: Elevated plus maze
F4/80: EGF-like module-containing mucin-like hormone receptor-like 1
FST: Forced swim test
GTT: Glucose tolerance testing
HFD: High-fat diet
IBA-1: Ionized calcium binding adaptor molecule 1
IDE: Insulin-degrading enzyme
IL-1β: Interleukin-1β
IL-6: Interleukin-6
IP: Intraperitoneal
LPL: Lipoprotein lipase
MHCII: Major histocompatibility complex class II
Pgk1: Phosphoglycerate kinase 1
RP: Retroperitoneal
SAB: Spontaneous alternation behavior
SDHA: Succinate dehydrogenase complex, subunit A, flavoprotein
SEM: Standard error of the mean
TLR4: Toll-like receptor 4
TNFα: Tumor necrosis factor α
Veh: Vehicle
