# Supplementary figures and images for: TLR4 inhibitor TAK-242 attenuates the adverse neural effects of diet-induced obesity
Source: J Neuroinflammation. 2018 Nov 5;15:306. doi: 10.1186/s12974-018-1340-0 (PMC6217784; doi:10.1186/s12974-018-1340-0)

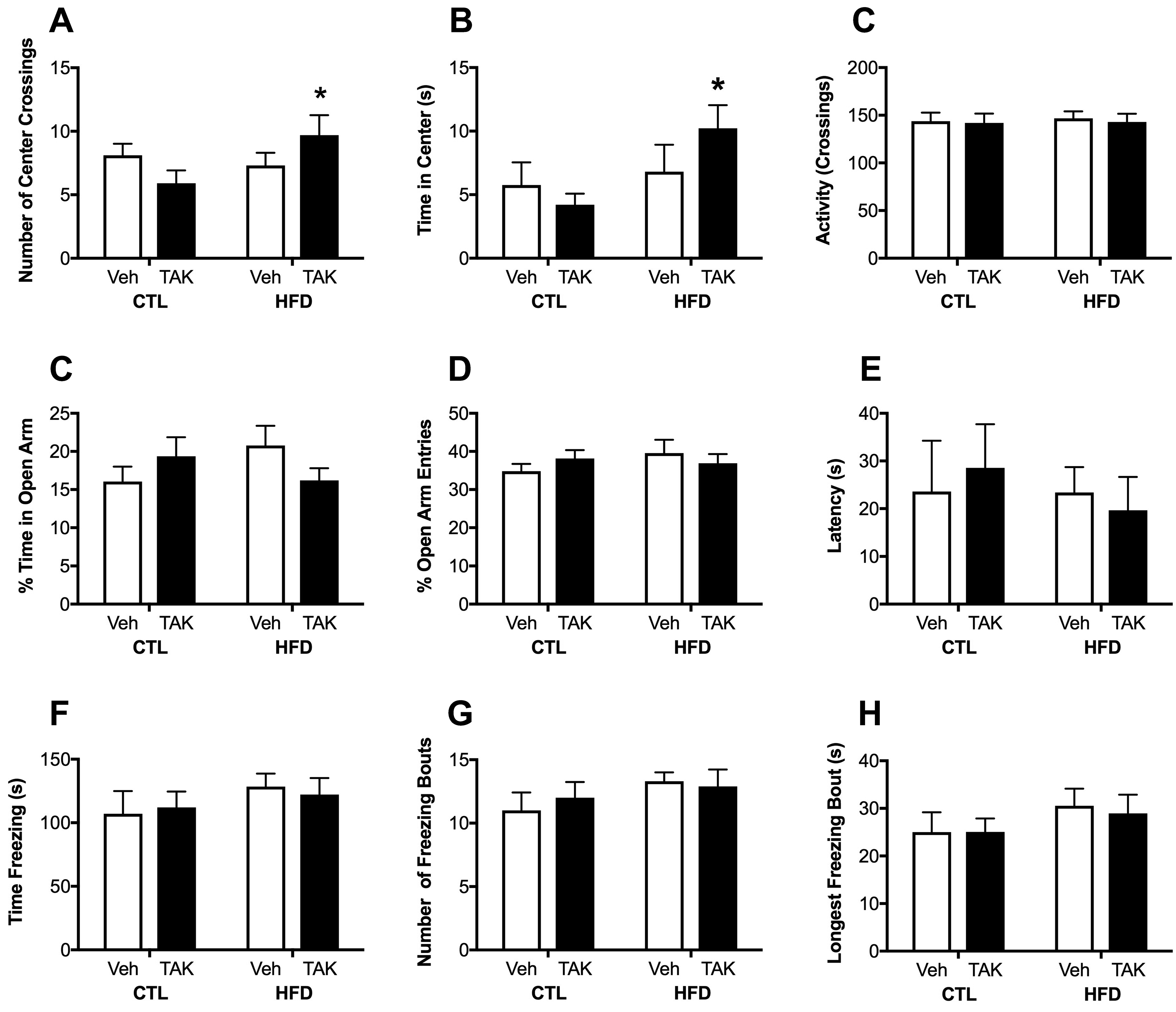

Supplement: Supplementary file 1 — Figure S1. Effects of diet and TLR4 inhibition on exploration, anxiety-like, and depressive-like behaviors. Exploration, anxiety-like, and depressive-like behaviors in control (CTL) and high-fat diet (HFD)-fed mice treated with vehicle (Veh) or the TLR4 inhibitor, TAK-242 (TAK). A-C) Explorative and anxiety-like behaviors were examined in the open field. A) The number of times animals entered the center square of the open field and B) the amount of time they spent in the center field. C) General locomotor activity as assessed by the total number of square crossings. D-F) Anxiety-like behavior was assessed in the elevated plus maze. D) The amount of time spent in the open arm of the maze, and E) the number of times the animals crossed into the open arm. F) The latency to enter the open arm for the first time. G-I) Depressive-like behaviors were examined in the forced swim test. G) The total amount of time the animals spent immobile and H) the number of times the animals were immobile. I) The length of the single longest time spent immobile. Vehicle-treated animals are shown in white bars and TAK-242-treated are shown in black bars. *p < 0.05 relative to drug treatment-matched mice in control diet condition. (TIFF 681 kb) [file 12974_2018_1340_MOESM1_ESM.tiff]
